# The Assessment of Grief in Refugees and Post-conflict Survivors: A Narrative Review of Etic and Emic Research

**DOI:** 10.3389/fpsyg.2018.01957

**Published:** 2018-10-22

**Authors:** Clare Killikelly, Susanna Bauer, Andreas Maercker

**Affiliations:** Division Psychopathology and Clinical Intervention, Department of Psychology, University of Zürich, Zürich, Switzerland

**Keywords:** narrative review, refugees, post conflict survivors, prolonged grief disorder, assessment, ICD-11

## Abstract

**Background:** Prolonged grief disorder (PGD) is a new mental health disorder that will be recognized by the World Health Organization’s disorder classification, the ICD-11, in 2018. Current assessment measures of PGD are largely based on North American and European conceptualizations of grief (etic i.e., from the perspective of the observer). However, research is emerging from communities outside of the Global North, in particular, conflict-exposed communities, exploring local models (emic i.e., from within the cultural group), assessment measures and symptoms of grief. Several reviews have found that refugees have higher rates of mental illness, defined by etic standards as depression, post-traumatic stress disorder (PTSD), anxiety disorders and psychotic symptoms. Yet, presently there are no reviews documenting the assessment of PGD in refugees and post conflict survivors.

**Method:** This narrative review will provide an overview of studies that assess grief in refugees to (1) identify current assessment measures of grief in refugees (i.e., type and frequency of questionnaires used, whether Global North-based, etic, or locally developed, emic, and the level of cultural adaptation) and (2) to document the variety and rate of grief symptoms identified with Global North standard measures and/or local measures (i.e., the endorsement of standard symptom items and the identification of culturally specific symptoms of grief).

**Results:** This review revealed 24 studies that assessed disordered grief in refugee or post conflict samples. Studies were heterogeneous in their assessment methods; the majority (*n* = 17) used an etic approach, four used a combined etic/emic approach, and three used a predominantly emic approach. The rate of disordered grief was high depending on cultural adaptation approach (31–76%) and when standard etic measures were used the disordered grief rate was 32%.

**Conclusion:** These findings will help to guide future studies to provide accurate assessment of grief in refugee and post conflict populations and has implications for improving cultural knowledge in clinical practice.

## Introduction

As the number of displaced people increases above 60 million (United Nations High Commissioner for Refugees UNHCR, 2017), researchers have mobilized to provide up to date and extensive knowledge on the physical and mental health of refugees, asylum seekers, displaced persons and forced migrants as well as post-conflict survivors. Several reviews have found that refugees have higher rates of mental disorders such as depression, post-traumatic stress disorder (PTSD), anxiety disorders (Fazel et al., 2005; Porter and Haslam, 2005) and psychotic symptoms compared to non-refugee migrants (Heeren et al., 2014; Hollander et al., 2016). Yet, there remains one mental health concern that is often not explicitly or consistently evaluated in refugees and post conflict survivors: prolonged grief disorder (PGD). Despite the knowledge that refugees are highly likely to experience severe and repeated exposure to violence, traumatic loss, abuse of human rights and to witness the death of family and friends (Chen et al., 2017; Miller and Rasmussen, 2017), presently there are no reviews documenting the assessment of PGD or disordered grief in refugees or post conflict survivor samples.

In societies of the Global North (i.e., formerly labeled as Western societies), PGD is an abnormal reaction to loss and is recently recognized as a new disorder in the ICD-11 diagnostic criteria (Maercker et al., 2013)—note: with a somewhat different concept from PGD described by Prigerson et al. (2009). Previously disordered grief was also identified as pathological grief, complicated grief, or in recent US research as persistent complex bereavement disorder (PCBD) (Wagner and Maercker, 2010); the DSM-5 included PCBD in the section of disorders requiring further study (American Psychiatric Association, 2013). Building on these previous definitions and conceptualizations, the ICD-11 criteria for PGD has been refined to be clinically useful, valid and reliable (Maercker et al., 2013; Maciejewski et al., 2016). The symptom structure was simplified to consist of two main symptoms related to longing and persistent preoccupation with the deceased, emotional distress, functional impairment and a consideration for different cultural norms and practices. For example, the criteria state that symptoms should be present for at least 6 months, however, this will defer to clinical opinion and cultural norms i.e., within the German context 1 year of mourning or ‘Trauerjahr’ is considered normal and this should be considered when assessing patients in the German context (Hays and Hendrix, 2008). Evidently, although these criteria were developed within the North American and European psychiatric context, the simplified structure and cultural caveats that aim to assess PGD across different populations, in particular refugees and post conflict samples, are essential for mobilizing support and providing effective treatment (Killikelly and Maercker, 2018). The construct validity (the ability to measure what is thought to be measured within the particular context) (Nunnally and Bernstein, 1994) of ICD-11 PGD criteria has yet to be validated in different cultural contexts. As stated by Lewis-Fernandez and Kleinman: ‘We cannot assume that an assessment measure developed for North America and European populations will be valid and accurate in a different context’ (Lewis-Fernandez and Kleinman, 1995).

Presently, it is unknown if the new ICD-11 PGD symptom structure defined above is applicable to refugee populations. Until now, the range of grief reactions in refugees, including differences and similarities with the North American and European based psychiatric criteria, have not been systematically documented. For many years there has been a reliance on assessment measures developed in North America and Europe. A review by Hollifield et al. (2002) found that 78% of studies relied on measures developed in the Global North to examine refugee mental health. We cannot assume that an assessment measure developed for North American and European populations will be valid and accurate in a different cultural context (Lewis-Fernandez and Kleinman, 1995; Summerfield, 1999). For example, several studies have documented grief and loss in refugees with varying prevalence rates: 41% of West Papuan refugees experienced the traumatic loss of a beloved family member (Tay et al., 2015); and the prevalence of PGD in refugees ranges from 8% among Rwandan war widows and orphans (Schaal et al., 2010) to 54% in resettled Bosnian refugees (Craig et al., 2008). The broad range of prevalence could indicate that symptoms of grief vary significantly between cultures, i.e., that different cultures have more severe symptoms of grief than others, or it could indicate that some assessment measures are more sensitive to capturing ‘true’ symptoms of grief in some cultures but not in others. Although there may be common psychopathological responses to distress a better understanding of specific culturally relevant symptoms may afford better communication, more effective mental health interventions and stronger alliance with health care professions (Summerfield, 1999; Aggarwal et al., 2014). Additionally, the culture of the health care setting may impact on the expression of grief symptoms. For example, Zhou et al. (2016) determined that in a group of Chinese outpatients with depression, the presence of somatic symptoms (e.g., headache) could indicate the felt bodily experience of depression, or it could be a more socially acceptable way of presenting distress. The inclusion of local meanings and culturally bound syndromes can improve the validity and reliability of assessment measures.

In cross-cultural psychology, the term *etic* is used when behaviors are studied from outside a culture with the goal of finding universal patterns, whereas *emic* refers to an approach where behaviors are studied from within a culture to understand the unique aspects of that phenomenon (Fernando, 2012). Alternatively formulated, *etic* research takes the perspective of an outside observer, in contrast to the *emic* perspective, from inside the culture (Rasmussen et al., 2014). The balance between etic and emic perspectives is often missing in current discussions, despite it being a crucial issue, not only for practitioners, but also for researchers (Fernando, 2012). Lewis-Fernandez and Kleinman (1995) assert that any culturally congruent psychiatric assessment should aim to blend *emic* or insider perspective with an *etic* perspective on mental health. Furthermore, the authors stated that the relationship between symptoms and illness is complex and that finding a superficial resemblance among symptom clusters across cultures does not guarantee that the same disorder is being validly identified.

Indeed, recent reviews have revealed unique and important findings through cultural adaptation using an emic or combined emic/etic approach. Rasmussen et al. (2014) reviewed 55 studies with data of 32 Non-European countries to investigate PTSD in emergency settings outside North America and Europe. In their review, the following explicit emic approaches in the 55 studies were used: unspecified ethnography, key informant interviews, ethnographic interviewing, surveys, free listing, participant observation, focus groups, clinical interviews and observation, life histories, presenting or eliciting clinical vignettes, and comparison tasks. The most notable result they found was the variety of cultural concepts of distress (CCD’s) associated with PTSD, e.g., “ataque de nervios” as panic-like disorder in Latino Caribbean (Lewis-Fernández et al., 2010) or spirit possession characterized by dissociation in African studies (Betancourt et al., 2009; Ventevogel et al., 2013). Contrary to previous assumptions that PTSD symptoms are universal, this review emphasizes the fact that there is substantial cross-cultural variation of posttraumatic symptom presentations and that future research should include emic literature to improve cultural validity.

In recent years, several authors have used and adapted North American and European measures to be used in different refugee populations. Currently there are no reviews of grief assessment, of either etic or emic methods, for refugees or post conflict survivors. This narrative review will review recent studies of grief in refugees and post-conflict survivors in terms of the type of approach to cultural adaptation (etic or emic), the unique culturally relevant symptoms of grief revealed and the rates of disordered grief identified across the different etic and emic approaches.

## Materials and Methods

### Study Design and Inclusion Criteria

In this narrative review, the following PICOS standards (Higgins and Green, 2008) for inclusion criteria were defined: (1) the sample consisted of adult (>18 years) refugees/migrants/asylum seekers/people living in a (post-) conflict zone. (2) The article has been published in a peer-reviewed journal and (3) the article’s language was English. Furthermore, (4) a measurement of grief symptoms was used.

### Search Strategy

**FIGURE 1:**
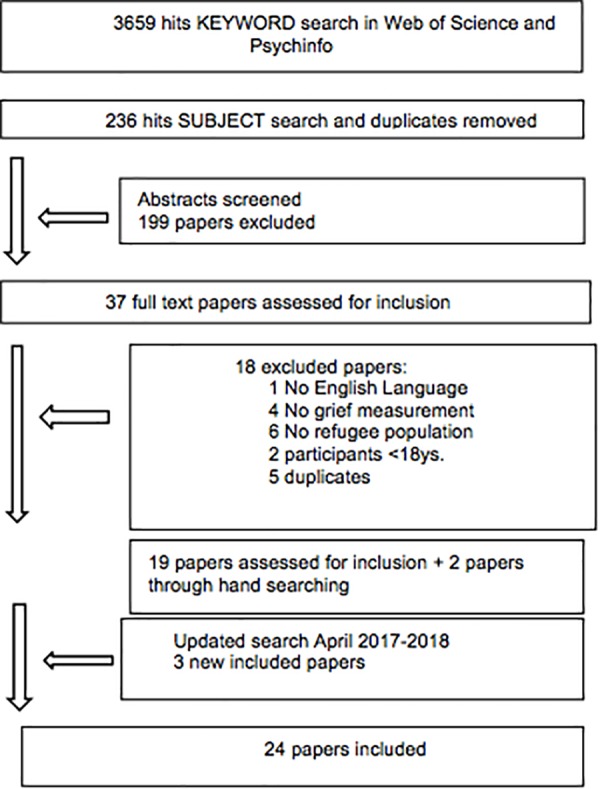
Flow chart of search strategy.

The search was conducted within the time frame 2000 until April 2018, on two separate subject specific databases for psychological material Web of Science and PsychINFO. As the aim of the review is to provide an up to date account of the state of the field papers before 2000 were not included. Web of Science and PsychINFO databases were chosen as they are major literary databases in the field of psychiatry and psychology. Keyword searches were executed with distinct combinations of search terms: (grief OR griev^∗^ OR bereave^∗^ OR loss OR mourn^∗^) AND (refuge^∗^ OR migrant^∗^ OR asylum seek^∗^). The selected search terms provided 2010 hits in Web of Science and 1649 hits in PsychInfo (Figure 1). To refine the search, the following limiters were used: no conference proceedings or books, research articles only, English language, adults over 18 (since child and adolescents grief expressions and symptoms vary greatly). A subject category search selecting the relevant subjects (excluding basic science categories, e.g., chemistry, physics, environmental sciences, mathematics, engineering) was conducted in Web of Science and PsychINFO. Duplicates were removed and the articles to be screened were reduced to 237 hits. CK and SB screened titles and abstracts according to the inclusion criteria. 200 papers, that did not meet the criteria, were excluded, resulting in 37 potentially relevant articles. Full texts were downloaded and reviewed. Eighteen were excluded. An update of the search was conducted from April 2017 until April 2018 and revealed three unique new articles to include. In total, 24 studies were included in the final analysis (see Figure 1).

### Data Extraction

**FIGURE 2:**
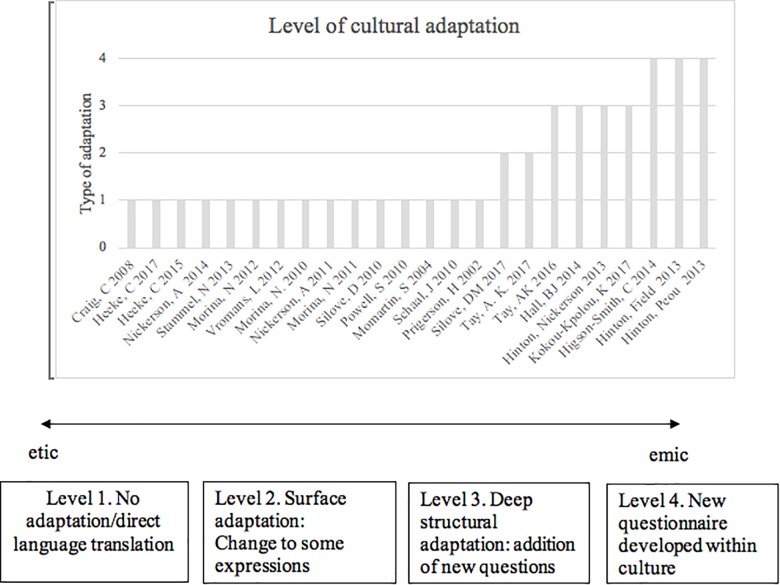
Level of cultural adaptation and continuum of included studies according to approach.

A standard form was used to extract data from selected studies to create two results tables (please see **Appendixes A**, **B**). The first results table (**Appendix A**) included information on the study characteristics including: study date, first author, study title, study country, description of population (refugees, displaced or people living in a conflict zone), population origin, sample size, male, female, gender distribution and country of residence. The second table (**Appendix B**) consists of time since loss, type of bereavement, grief measure, rates of disordered grief, cultural specific adaptation and psychometric properties. All included articles were read thoroughly and systematically screened for the above data. If there were several publications of the same study all publications were included if they used different assessment measures or methods for cultural adaptation of grief measures. Additionally, studies were coded for the cultural specific symptoms of grief and the level of cultural adaptation. The level of cultural adaptation was assessed using a standard form with criteria developed and amended following a continuum for developing culturally appropriate interventions (Okamoto et al., 2014). Four levels of adaptation were coded (1) no adaptation, but direct translation using standard translation practices, including consultation with locals or experts (2) surface adaptation: small changes are made to the structure or content of the original questionnaire or interview for the purposes of including some cultural expressions, idioms or beliefs within the original questionnaire, methods including field testing and piloting (3) deep structural adaptation: use of systematic methods to develop culturally appropriate questions and content in addition to the original questionnaire, e.g., the inclusion of new unique content or questions, methods include interviews and focus groups (4) Culturally grounded adaptation: the development and refinement of a unique questionnaire specifically tailored to a particular cultural group (see Figure 2). Coding was completed by both CK and SB. Disagreement between researchers was dealt with by consensus with a senior member of the research team (AM).

## Results

### Study Characteristics

A total of *n* = 8600 individuals participated in the included studies (see Table 1), 43% males (*n* = 3701) and 57% females (*n* = 4899). The mean sample size was *M* = 358 participants. The smallest sample consisted of *n* = 60 and the largest of *n* = 2964 participants. The mean age across all studies was *M* = 42 years, with a range from 18 – 80 years. When indicated, the mean time since loss was *M* = 14.2 years, with a range from 6 months to more than 45 years. Data were obtained from 12 different countries including samples from Africa (*n* = 3), Americas (*n* = 2), Eastern Europe (*n* = 7), Asia (*n* = 8), Middle East (*n* = 4). Samples included refugees (*n* = 10), post conflict survivors (*n* = 9), internally displaced people (*n* = 2), or a combination of migrants, refugees and internally displaced people (*n* = 3).

**Table 1:** Demographic characteristics of study participants across 24 included studies.

		*N*	%
Number of bereaved	Total	8600	
Gender	Male	3701	43
	Female	4899	57
Type of Loss per study	Traumatic/conflict related	24	100
	Sickness/health	5	22
	Disappearance, unknown	3	13
	Disaster/Accident	3	13

		***M***	**Range**

Mean sample size		358	60–2964
Mean age (years)		42	18–80
Mean time since loss (years)		14.2	0.5 to >45

All of the 24 included studies measured traumatic or conflict-related loss. In addition, several studies also measured sickness or health-related loss (5), disappearance or unknown loss (3) and disaster or accident related loss (3). This exemplifies that the majority of measurement is focused on traumatic and conflict related loss in refugee and post conflict survivor samples (see Table 1).

### Approach to Grief Measurement

The methodological approach to measuring grief varied along the *etic-emic* continuum described above. Fifteen studies used a direct translation approach, two studies conducted surface adaptation, four studies conducted deep structural adaptation and three studies conducted culturally grounded adaptation (see Table 2). The following sections synthesize the results in terms of the three main approaches used to assess grief: predominantly etic (direct translation and surface adaptation), combined emic/etic (deep structural adaptation) and predominantly emic (culturally grounded) approaches. The main findings in terms of rates of disordered grief are presented for each approach.

**Table 2:** Cultural specific items revealed from emic deep structural and grounded adaptations.

First Author, Year	Culturally specific item	Rate of grief-related distress	Level of adaptation
Tay et al., 2016	‘Duka Cita’ or grief reaction in Bahasa Indonesian	38.7% strong feelings of yearning/longing	Deep structural
Hall et al., 2014	the Kurdish local population, ‘Imitating behaviors of someone who had died’	Mean scores on adapted ITG were 8.89/36	Deep structural
Hinton et al., 2013b	Cambodian Khmer language the term ‘nuk sreunoh’: to recall with nostalgic longing and CSM-G; ‘In this last month when you thought about the deceased, how much did it cause you to feel not well in your mind or body?	PG-13 endorsed by 8%, CSM-G endorsed by 31%	Deep structural
Kokou-Kpolou et al., 2017	Feelings of guilt related to the context of the death of the loved one and open ended questions about reasons for guilt feelings.	Mean scores on the ICG-R were for migrants and refugees 31.33 vs. 40.20 respectively. Scores on the additional grief items were the most highly endorsed items for both refugees and migrants (mean 2.85 and 2.15 out of 4 respectively).	Deep structural
Higson-Smith, 2014	Bereaved clients (compared to non bereaved) were more likely to experience elevated symptoms of distress such as crying easily, suicidal thoughts, pounding heart and headaches.		Grounded
Hinton et al., 2013c	(1) tdaay haong: or bad death (2) rebirth: (3) dreams of the dead ‘khyal attack’: distress and anxiety type somatic symptoms	76% pained remembering of the deceased.72% concerns over spiritual status of the dead, 73% cried when recalling the dead, multiple somatic symptoms from 67 to 88%, 70% of participants attributed distress trigged by pained recall of the deceased to a *khyal attack*	Grounded
Hinton et al., 2013a	Dreams of the dead questionnaire	52% of participants had dreamed of the deceased in the past month. The frequency of dreams was significantly correlated with scores on the PG-13 (*r* = 0.59)	Grounded

*PG-13, Prolonged Grief Disorder scale; ITG, Inventory of Traumatic Grief; ICG-r, Inventory of Complicated Grief-revised; CSM-G, Culturally Specific Measurement Grief.*

#### Approach 1: Predominantly Etic

**FIGURE 3:**
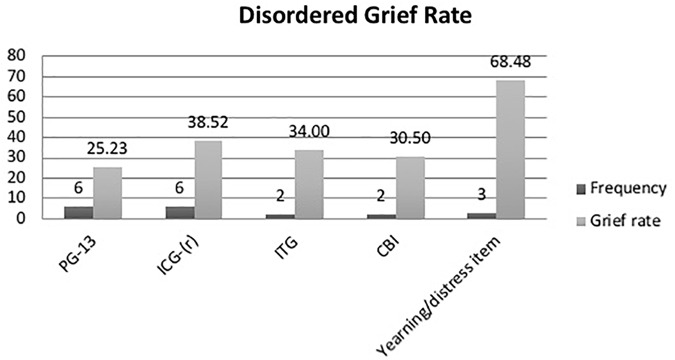
Rate of disordered grief and frequency of questionnaire use for etic studies. Prolonged Grief Disorder scale (PG-13); Inventory of Complicated Grief and the Inventory of Complicated Grief-Revised (ICG/ICG-R); Inventory of Traumatic Grief (ITG), Core Bereavement Items (CBI). The yearning/distress item was derived from data from Vromans et al. (2012),Silove et al. (2017), and Tay et al. (2017) who examined the rate of endorsement of single items of yearning or loss distress on standard grief measures (e.g., ICD-11 and idiosyncratic grief questionnaire) which were deemed acceptable in the local community.

The majority of the studies used standard grief questionnaires, developed in the Global North, to measure disordered grief in refugee populations (Prigerson et al., 2002; Momartin et al., 2004; Craig et al., 2008; Morina et al., 2010, 2011; Powell et al., 2010; Schaal et al., 2010; Silove et al., 2010, 2017; Nickerson et al., 2011, 2014; Morina and Emmelkamp, 2012; Vromans et al., 2012; Stammel et al., 2013; Heeke et al., 2015, 2017; Tay et al., 2017). Data was collected using the Prolonged Grief Disorder scale, PG-13 (Prigerson et al., 2009) (*n* = 6), the Inventory of Complicated Grief and the Inventory of Complicated Grief-Revised, (ICG/ICG-R) (Prigerson et al., 1995; Prigerson and Jacobs, 2001) (*n* = 7), the Inventory of Traumatic Grief (ITG) (HG. Prigerson et al., 1999) (*n* = 2), and the Core Bereavement Items (CBI) (*n* = 2) (Burnett et al., 1997). Where reported, the rates of disordered grief varied across these standard assessment measures with an average of 32% (range 8–69) of participants scoring in the disordered or severe range (see Figure 3). Additionally one study used an idiosyncratic Grief Questionnaire to assess refugees from Burma in Australia (Steel et al., 2007 in Vromans et al., 2012) and one study the UCLA Grief Inventory (Saltzman et al., 2001 in Powell et al., 2010) (*n* = 1). Vromans et al. (2012) found that 98% of participants endorsed interpersonal grief (mean of 2.97 measured on a 5 point scale) and 58% of participants reported loss distress. In Powell et al. (2010b), the UCLA Expanded Grief Inventory found mean scores of 3.03 and 2.40 for existential and traumatic grief respectively.

In terms of cultural adaptation several studies (*n* = 15) used previously translated measures or followed a standard protocol for translation including forward and back translation along with consultation with local participants and experts (e.g., Bontempo, 1993). Two studies conducted surface cultural adaptation. For example, after the direct translation of the questionnaire, Silove et al. (2017) field-tested a pool of items from the ICD-11 PGD criteria (Maercker et al., 2013) in a community sample of Timor-Leste locals. Four items were determined to be most common and understandable in the community. These were assessed in a sample of 677 couple-dyads (*n* = 1354 individuals): persistent yearning/longing (endorsed by 73.95% of participants), bitterness about the death (endorsed by 44.55%), feelings of emptiness (endorsed by 40.65%) and functional impairment (endorsed by 65.8%). A follow up study (Tay et al., 2017) followed the same surface cultural adaptation procedure and found similar rates for *n* = 2964 individuals: persistent yearnings or longing for the deceased (endorsed by 73.5%), feelings of bitterness about the death (43.6%), and feelings of emptiness (38.9%), functional impairment associated with grief symptoms (32.3).

As mentioned above Vromans et al. (2012); Silove et al. (2017), and Tay et al. (2017) examined the rate of endorsement of single items of yearning or loss distress on standard grief measures (e.g., ICD-11 and idiosyncratic grief questionnaire) which were deemed acceptable in the local community. The percent of participants endorsing these specific items, here labeled yearning/distress item, is substantially high (68.4%).

#### Approach 2: Etic/Emic

Four studies utilized a combined etic/emic approach by significantly amending or adding to the structure and content of a standard grief measure to reflect culturally specific symptoms of grief (Hinton et al., 2013b; Hall et al., 2014; Tay et al., 2016; Kokou-Kpolou et al., 2017). Also referred to in this review as deep structural adaptation, Tay et al. (2016), conducted focus groups with West Papua refugees and local psychiatrists to determine the acceptability and comprehensibility of symptom items of the proposed ICD 11 criteria for PGD and the PCBD DSM-5 criteria. The construct of disordered grief was identified to strongly correspond to the indigenous construct ‘Duka Cita’ or *grief reaction* in Bahasa Indonesian. Along with the PGD and PCBD symptom item list, consultation with locals also revealed other commonly endorsed symptoms including confusion, diminished sense of identity and difficulties planning for the future, the latter two also appear in the PCBD disorder definition. These were subsequently added to the grief measure. This resulted in an 18 item grief measure with endorsements ranging from 38.7% for strong feelings of yearning/longing for the person who is dead, to 5.2% for difficulty or been reluctant to plan for the future.

Hall et al. (2014) used an established protocol for cultural adaptation developed by Bolton (2008). A mixed methods approach was used; qualitative interviews were conducted with the local Kurdistan population to identify culturally specific symptoms of grief. This was assessed alongside the items of the Inventory of traumatic grief (Prigerson et al., 1999). This method resulted in a 12-item traumatic grief scale with 11 items from the ITG and one symptom specific to the Kurdish local population, ‘Imitating behaviors of someone who had died.’ Mean scores on this measure were 8.89/36.

Hinton et al. (2013a,b,c) conducted a series of studies examining grief and trauma in a refugee community in Cambodia. After translation into the local Khmer language the term ‘nuk sreunoh’ meaning to recall with nostalgic longing, was used to define the index death. The PG-13 (Prigerson et al., 2008) was used to assess symptoms of grief with an important addendum (Hinton et al., 2013b). Based on previous research (Hinton et al., 2009) by the authors ‘concerns about the deceased not yet being reborn’ were added to the scale. Additionally, participants completed a culturally sensitive measure of grief related distress (CSM-G); ‘In this last month when you thought about the deceased, how much did it cause you to feel not well in your mind or body?’

This item was developed based on the first authors clinical experience with Cambodian refugees through extensive interviews prior to the study. 8% of the refugees were found to meet criteria for PGD based on the PG-13, however 31% of the sample scored in the severe range (4/5) on the CSM-G.

After a preliminary survey of the refugee community in France, (Kokou-Kpolou et al., 2017) added two questions to the standard grief measure ICG-R (Prigerson et al., 1995; Prigerson and Jacobs, 2001). The first question was feelings of guilt related to the context of the death of the loved one and the second question was open ended and inquired about reasons for guilt feelings. Additionally, they developed an idiosyncratic measure of ‘Death and Ritual information.’ Participants were asked about funeral rites, ceremonies, repatriation of the body, participation in rituals and other cultural ceremonies. Mean scores on the ICG-R were compared for migrants and refugees (31.33 vs 40.20 respectively). Scores on the additional grief items were the most highly endorsed items for both refugees and migrants (mean 2.85 and 2.15 out of 4 respectively). Additionally, for those who participated in bereavement rituals their scores were significantly lower on the ICG-r.

#### Approach 3: Predominantly Emic

Few studies (*n* = 3) employed a predominantly emic approach. Higson-Smith (2014) conducted a mixed methods approach and reviewed data from case files of 85 torture survivors in Sub-Saharan Africa. A thematic analysis on the content of the case files was conducted to collect quantitative and qualitative data on the mental health and trauma history of help-seeking patients in a local treatment center. It emerged from the review that sudden and violent bereavement was very common and symptoms of disordered grief frequently reported. Bereaved clients (compared to non-bereaved) were more likely to experience elevated symptoms of distress such as crying easily, suicidal thoughts, pounding heart and headaches. Additionally, semi-structured interviews with 14 torture survivors revealed that bereavement was a key concern for participants. The results of the qualitative interviews revealed key findings relevant to the assessment of disordered grief; for those who entered treatment 1 year after bereavement they had significantly worse distress on all symptoms compared to those with earlier treatment; yearning for the deceased was a common theme along with social and emotional stressors, and the moral responsibility that clients might feel should be openly discussed.

Hinton et al. (2013c) conducted semi-structured interviews to examine spiritual beliefs about death and rebirth in a group of Cambodian refugees. The interviews revealed key aspects for a new ontology of bereavement for this cultural group including (1) tdaay haong: or bad death, a traditional belief that a violent death may indicate a past ‘demerit’ and prevent rebirth, (2) rebirth: is an essential component of the Cambodian belief system and those who died violently may not be reborn but wander in a purgatory state, (3) dreams of the dead are indicators of the person has not been reborn, especially 1 year after the death. Based on these factors a bereavement questionnaire was developed to assess the following: painful recall of the loved one, self-perceived severity of pained recall, frequency of pained recall, dreams and pained recall, spiritual concerns, crying during pained recall, somatic distress and pained recall, trauma and pained recall, culturally specifically labeling and treatment of episodes of pained recall (Hinton et al., 2013c). A specific cultural interpretation of distress ‘*khyal attack’* was also assessed (Hinton et al., 2013c). This has been translated as wind attack and refers to the belief that distress and anxiety type somatic symptoms are caused by a disruption of the flow of *khyal* which is a wind-like substance that flows through the body with the blood. When severe, these attacks can cause death and are treated by placing coins in ‘wind oil’ and pushing these on the skin. Results from the culturally grounded questionnaire indicated that 76% of participants experienced pained remembering of the deceased. In terms of other cultural specific factors, 72% had concerns over the spiritual status of the dead, 73% cried when recalling the dead, along with multiple somatic symptoms experienced to high degree ranging from 67–88% (e.g., blurry vision, palpitations respectively) depending on the symptom. Past trauma was brought to mind in 90% of participants and 70% of participants attributed distress trigged by pained recall of the deceased to a *khyal attack*. 78% of participants did coining to treat these attacks. Based on these findings the centrality of dreams to bereavement for Cambodian refugees was assessed. An idiosyncratic dream measure was developed to assess the frequency of dreams, who was dreamed about, types of dreams and the spiritual state of the deceased. It was found that most dreams (80%) were about someone who had died in the conflict and 25% involved trauma, 13% were nostalgic and 62% were a visitation. This scale was assessed in an additional study (Hinton et al., 2013a). 52% of participants had dreamed of the deceased in the past month. The frequency of dreams was significantly correlated with scores on the PG-13 (*r* = 0.59).

## Discussion

This narrative review revealed three main findings. Firstly, three different approaches to grief assessment were documented; predominantly etic, combined emic/etic, and predominantly emic. Secondly, the rate of disordered grief or PGD varied depending on the type of measure used, yet overall rates of PGD were high across standard etic measures (32%). Thirdly, deep structural and grounded cultural adaptation occurred in seven studies. This revealed unique culturally specific symptoms of grief and higher rates of grief symptoms on these symptoms (ranging from 31 to 76%). Our findings support previous reviews that show important cross-cultural differences in symptomatology for other stress related disorders. Hinton and Lewis-Fernández (2011) and Rasmussen et al. (2014) found ‘substantial cross cultural variation’ in PTSD symptomatology. Here we find important variations in assessment measures and symptoms of grief across both emic and etic research methodologies.

Despite variability in the types of etic questionnaires used, the rate of disordered grief is high for refugee and post conflict samples. A pooled rate of 32% of participants scored in the severe or disordered range. This is much higher than prevalence studies of general population samples. For example, recent studies of the rates of disordered grief in the Global North find prevalence rates ranging from 3.7% (Kersting et al., 2011) to 9.8% (Lundorff et al., 2017). The current high rate of disordered grief in refugee and post conflict survivor samples is supported by previous findings that war-related bereavement/traumatic bereavement result in higher risk of disorder. Several reviews of refugee health have consistently found higher rates of distress and disorder across both physical and mental health conditions (Fazel et al., 2005; Porter and Haslam, 2005). The largest review of 81,866 refugees found rates of 30% for depression and PTSD in refugee populations (Steel et al., 2009). The strongest predictors of disorder were the number of traumatic events experienced and exposure to torture. In a representative population-based German sample, those bereaved by a violent death had a disordered grief rate of 20% (Kersting et al., 2011). In the current review all 24 studies examined grief in the context of trauma or conflict related loss, it is therefore not surprising to find a similarly high rate of disordered grief.

This raises the important issue of ‘normality’ and what defines the distinction between a pathological response and a normal response within a cultural context. Normality could be defined quantitatively, e.g., if a high number of the sample experience these symptoms, then perhaps this is a cultural norm, however we would argue that clinical opinion and the context of the symptoms should be taken into account. The finding that 68% of the refugee samples experienced intense yearning and distress could indicate that this is a ‘norm,’ however, when considering the context violence and trauma and the implications it is more likely that this high number reflects the multiple traumas experienced by these individuals within this specific post-conflict context and should not be considered a ‘norm.’

Refugees experience many losses, not only the loss of a loved one, but also the loss of homeland, cultural group, employment, housing, and security. It is to be expected that being bereaved is only one of many losses that may impact on mental health. The Multidimensional Loss Scale (MLS) (Vromans et al., 2012) is one of the first instruments designed specifically to index *Experience of Loss Events* and *Loss Distress* across multiple domains (cultural, social, material, and intrapersonal) relevant to refugee settlement. Assessed in recently settled Burmese adult refugees (*N* = 70) the scale has five dimensions of loss: Loss of Symbolic Self, Loss of Interdependence, Loss of Home, Interpersonal Loss, and Loss of Intrapersonal Integrity. This provides a promising framework for documenting the multiple losses and wider context of grief in refugees. Similarly, Miller and Rasmussen (2017) propose an ecological model of refugee distress. They assert that conflict-related loss and trauma are not the only events that can precipitate a mental health disorder. There are several post-migration stressors such as loss of social networks, isolation, unemployment, poverty, discrimination and lack of basic resources and safety that may have a significant effect on mental health (Miller and Rasmussen, 2017). As identified by Hinton et al. (2013c) and Kokou-Kpolou et al. (2017), the inability to perform culturally specific death rites and rituals post-migration was associated with higher severity of grief symptoms. The socio-cultural context of post-migration and resettlement may provide a backdrop that may hinder or help recovery from conflict-related bereavement and loss.

The level of cultural adaptation varied with few studies using a combined emic-etic approach (*n* = 4) or a culturally grounded emic approach (*n* = 4). However, when these approaches were used culturally unique and meaningful symptoms were revealed. The emic adaptations pointed to three new symptom categories that should be considered in future cross-cultural grief assessment; somatic symptoms, spiritual concerns and dreams or re-experiencing the deceased. The one-item scale CSM-G (Hinton et al., 2013b) and the “khyal attack”-item (Hinton et al., 2013c) measured physical discomfort associated with grief. These items were well understood by participants as a culturally sensitive way of determining the extent of self-perceived severity of grief. Cross-cultural research indicates that physical symptoms (e.g., palpitations, headaches and tinnitus, etc.) are often mentioned when assessing disordered grief (Hinton et al., 2013c). Physical symptoms are also endorsed in studies conducted with non-refugee populations in the Global North. Several studies have reported a greater occurrence of physical health complaints in bereaved people (compared with matched controls), ranging from physical symptoms such as headaches, dizziness, indigestion and chest pain (Stroebe et al., 2007). Interestingly, a recent study presented a phenomenon identified as ‘Ulysses syndrome’ or the psychosomatic disorder of modern migrants (Bianucci et al., 2017). The authors describe how migrants experience multiple stressors and this can lead to debilitating somatic complaints such as irritability, headache, migraine, nervousness, insomnia, fear and general discomfort. These findings and the culturally specific somatic items identified in this review point to the fact that somatic symptoms are not overtly part of –or are at least underrepresented– in the most standard grief measures. If somatic symptoms continue to be excluded from assessment measures this could lead to the mislabelling and misidentification of common and recognizable of mental health disorders such as depression, PTSD and PGD.

This underrepresentation is similar for spiritual concerns. Spiritual beliefs, such as ‘rebirth concerns’ (Hinton et al., 2013b) may be more important for mental health outcomes than previously assumed. For instance, religious beliefs can foster resilience during bereavement both by providing a stable, shared belief system and by providing affiliation and social support from the religious community (Bonanno et al., 2002). Furthermore, dreaming of the deceased was found to be an important indicator of grief symptoms in a Cambodian refugee population; it is strongly connected to spiritual beliefs about rebirth and reincarnation after death (Hinton et al., 2013a,b). A typology of dreams was developed as the type of dream (i.e., trauma reliving or visitation dream) may indicate the severity of grief distress. Indeed, the frequency of dreams was significantly correlated with PGD severity. Dreaming of the deceased seems to be a universal part of the grieving process and dreaming of the loved person in a positive state can be comforting for the bereaved (Kübler-Ross and Kessler, 2005). It is therefore imaginable that positive dreams of the deceased reduce the risk of developing prolonged grief symptoms or indicate adaptive recovery while dreaming of the deceased suffering is likely to produce negative feelings in the dreamer and add to the risk of disordered grief development.

In Global North samples, dreaming of the deceased was originally one of the 30 symptoms proposed as a key intrusive symptom of complicated grief (Horowitz et al., 1997), but is not used in the more recent Inventory of Complicated Grief (Prigerson et al., 1995). A study in a German sample found that dreaming of the deceased had poor ability to identify people with complicated grief and the authors suggested it should be dropped from the criteria (Langner and Maercker, 2005). However the ICG has other intrusive items such as “I hear the voice of the person who died speak to me” or “I see the person who died stand before me” and attests to the importance of assessing some form of re-experience of the deceased, whether in dreams, voices or hallucinations. As several authors have attested, grief can come with a strong desire to continue the bonds with the deceased (Currier et al., 2015). Hallucinations and visions of the deceased person are often experienced in both Western and Eastern populations (Zisook and Shear, 2009; Shimizu et al., 2017) and many take comfort in retaining objects or carrying on the legacy of the one who has died. “Imitating some of the same behaviors or characteristics of people who have died” was an item found to be specific for a Kurdish sample (Hall et al., 2014). It would be interesting to assess this symptom in a broad variety of cultures. In modern psychology, imitating the behaviors or characteristics of deceased loved ones can be seen as maladaptive yet has also been seen as a normal stage of “looking for the deceased followed by separating” of Kast’s four stage model of normal grief (Kast, 1982). This again, could be a form of re-experiencing the deceased that should be taken into account in the assessment of disordered grief. It may be important to clarify whether dreams and re-experiencing the dead is an important indicator of distress across cultural groups, especially in those cultures where dreaming is perceived to have specific and elaborate cultural meaning.

This review illustrates that there is substantial global variety among conceptualizations of what constitutes grief and what symptom endorsements it entails. The findings suggest that physical and spiritual symptoms should be a more familiar theme within the research of disordered grief. This should be undertaken on one hand for improved cultural sensitivity; on the other hand, a more comprehensive approach of symptom endorsements might also be applicable for Global North populations. In any case, future research should explore a broader variety of grief symptoms with a focus on cultural sensitivity. Physical symptoms should be assessed as well as patients’ religious/spiritual beliefs and the nature of their beliefs (e.g., belief in an afterlife), dreams (e.g., nightmares of deceased loved ones) or philosophical belief systems (e.g., believing in a just world) and re-experiencing the dead (Bonanno et al., 2002; Hinton et al., 2013a). The presence or absence of some of these symptoms and belief systems could become additional valid predictors of disordered grief vulnerability.

There are several studies that have attempted to develop cross-cultural adaptation guidelines and quality assessment tools, however, most of these apply to intervention studies. For example, Harper Shehadeh et al. (2016) conducted a systematic review and meta-analysis to evaluate the extent of cultural adaptation and the effectiveness of interventions for common mental health disorders. They developed a questionnaire to assess the level of adaptation for each study. This included questions such as ‘did you translate the intervention to local language?’, ‘did you consider metaphors in the cultural adaptation of your intervention?’ They found that the level of adaptation had a positive effect on the effectiveness of the intervention. The UN Inter Agency Standing Committee Guidelines on Mental Health and Psychosocial Support in Emergency Settings states that health care workers must not use assessment measures that are not validated for the local context. Presently several research groups have developed cross cultural assessment measures for mental health, including the Cultural Assessment Tools for Mental Health and Cultural Assessment from McGill University (Kirmayer et al., 2014), the Cultural Formulation Interview for the DSM-5 (Aggarwal et al., 2014), the Harvard Trauma Questionnaire (Mollica et al., 1992), and guidance for Culturally sensitive Assessment of Trauma (Good and Hinton, 2015). These research assessment tools should be adapted to include items relevant to disordered grief. Indeed both the DSM-5 and ICD-11 disorder definitions now include cultural caveats such as ‘the duration and severity of the grief response must exceed expected socio-cultural norms.’ Both of these disorder definitions provide a starting point for cross cultural considerations and comparisons.

### Strengths and Limitations

This is the first review of the assessment methods and rates of disordered grief in refugee post conflict survivor populations. It provides a comprehensive overview of published articles having used a valid grief measure on adult bereaved refugees. However, there are several noteworthy limitations to this review.

By only including English articles from peer-reviewed journals, valuable emic literature might have been excluded. As we did not use search terms in other languages we did not find and subsequently exclude any studies based on language. In the future, researchers should conduct the initial literature search using terms in several different languages, in order to capture important literature from around the world. This will provide a broader pool of research from which we may find more emic research. The current study found very few emic studies and the findings are limited to specific refugee groups such as West Papua and Cambodia. Given the recent influx of refugees from the Middle East, including Iraq, Afghanistan and Syria future research should focus on grief related distress in these refugee groups.

The second limitation concerns the time criterion. In order to diagnose PGD, at least 6 months since the loss have to have passed. Not all selected articles provided an exact time since loss, but this was counterbalanced by the time criterion that was, in most cases, inherent in grief instruments applied. Thirdly, this review did not assess the relationship to the deceased, which could have provided valuable information about predictors of severity of grief response. Only 14 out of 24 included articles indicated a disordered grief rate, which made it difficult to find accurate pooled PGD rates. This review benefits from an up to date overview of the most relevant and noteworthy papers in the field. In addition we provide a framework for future assessment of cross-cultural research along the etic/emic continuum. This could provide the foundations for further combined etic-emic research in cross-cultural populations.

### Future Research and Implications

As research and clinical practice involving refugee and migrant populations remains a pressing issue, future researchers and clinicians should use a combined etic/emic approach for grief assessment that is culturally valid and clinically sufficient. There is increasing recognition for the importance of including cultural concepts of distress or idioms of distress within the format of North American and European mental health assessments (Rasmussen et al., 2014). In summary, this is the first narrative review to evaluate grief measures and rates of disordered grief in the refugee population. The results indicate the assessment of PGD to be methodically heterogeneous, which makes it difficult to compare results. Future research should aim at methodological alignment and always provide a rate of disordered grief. The implementation of the suggested criteria for PGD for the new ICD-11, particularly in terms of the new socio-cultural caveats (Maercker et al., 2013), could aid in this process of cross cultural knowledge sharing and alignment.

## Author Contributions

CK conceptualized the main content, conducted the literature search, analysis, and wrote the main body of the manuscript. SB conducted the literature search and wrote parts of the manuscript. AM conceptualized the content of the manuscript and edited the manuscript.

## Conflict of Interest Statement

AM is chair of the work group on disorders specifically associated with stress in ICD-11 development at WHO. The views expressed reflect the opinions of the authors and not necessarily the Working Group and the content of this article does not represent WHO policy. The results of this paper are partially based on the Master’s thesis of SB. The remaining author declares that the research was conducted in the absence of any commercial or financial relationships that could be construed as a potential conflict of interest.
