# Supplementary material for: The Assessment of Grief in Refugees and Post-conflict Survivors: A Narrative Review of Etic and Emic Research
Source: Front Psychol. 2018 Oct 22;9:1957. doi: 10.3389/fpsyg.2018.01957 (PMC6204364; doi:10.3389/fpsyg.2018.01957)
Supplement: Supplementary file 1 [file Table_1.docx]

| **Date** | **First author** | **Study title** | **Country** | **Refugee population? (yes/no)** | **Population origin** | **Sample Size** | **Gender (%, f=female, m=male)** | **Age (average years)** |
| --- | --- | --- | --- | --- | --- | --- | --- | --- |
| 2017 | Kokou-Kpolou, K | A Cross-Cultural Approach to Complicated Grief Reactions Among Togo-Western African Immigrants in Europe | France | Togo West African migrants and refugees | Togo West Africa migrants and refugees | 74 | f: 37.8, m: 62.2 | 37.12 |
| 2017 | Tay, A. K. | The role of grief symptoms and a sense of injustice in the pathways to post-traumatic stress symptoms in post-conflict Timor-Leste | Australia | No,but living in post-conflict zone | Timor Leste | 2964 | F:51, M:49 | 36.4 |
| 2017 | Heeke, C | Conflict-related trauma and bereavement: exploring differential symptom profiles of prolonged grief and posttraumatic stress disorder | Germany | Yes,internally displaced | Colombia | 308 | m: 38,3 f: 61,7 | 48.5 |
| 2017 | Silove, DM | Symptoms of post-traumatic stress disorder, severe psychological distress, explosive anger and grief amongst partners of survivors of high levels of trauma in post-conflict Timor-Leste | Australia | No, but living in post-conflict zone | Timor-Leste | 1354 | m:50 f:50 | m:43,5 f:38,9 |
| 2016 | Tay, AK | Factorial structure of complicated grief: associations with loss-related traumatic events and psychosocial impacts of mass conflict amongst West Papuan refugees | Australia | Yes, but within same country | Papua New Guinea | 230 | m:137 (59.5 ) f: 93 (40.4 ) | 37 |
| 2015 | Heeke, C | When hope and grief intersect: Rates and risks of prolonged grief disorder among bereaved individuals and relatives of disappeared persons in Colombia | Germany | No, but living in a post-conflict zone | Columbia | 295 | f: 181 (61.4) m:114 (38.6) | m:48,6 |
| 2014 | Higson-Smith, C | Complicated Grief in Help-Seeking Torture Survivors in Sub-Saharan African Contexts | USA | Yes | Various African countries | 85 | m:39 f:46 | 33.9 |
| 2014 | Hall, BJ | A Longitudinal Investigation of Changes to Social Resources Associated with Psychological Distress Among Kurdish Torture Survivors Living in Northern Iraq | USA | Residing in conflict zone | Iraq | 96 | m: 52,6 f: 47,4 | 41.9 |
| 2014 | Nickerson, A | Posttraumatic stress disorder and prolonged grief in refugees exposed to trauma and loss | Australia | Yes | Iraq | 248 | m:48 f:52 | m:38.3 |
| 2013c | Hinton, Peou | Normal Grief and Complicated Bereavement Among Traumatized Cambodian Refugees: Cultural Context and the Central Role of Dreams of the Dead | USA | Yes | Cambodia | 100 | m: 35 f: 65 | 54,2 |
| 2013b | Hinton, Nickerson | Prolonged Grief in Cambodian Refugees Following Genocide: Rebirth Concerns and Avoidance of Reminders | USA | Yes | Cambodia | 100 | m:35+f:65 | 54.2 |
| 2013a | Hinton, Field | Dreams of the Dead Among Cambodian Refugees: Frequency, Phenomenology, and Relationship to Complicated Grief and Posttraumatic Stress Disorder | USA | Yes | Cambodia | 100 | f: 65 m:35 | 54,2 |
| 2013 | Stammel, N | Prolonged grief disorder three decades post loss in survivors of the Khmer Rouge regime in Cambodia | Germany | No, but living in a post-conflict zone | Cambodia | 775 | f:498 (64.3)m: 277 (35.7) M= 56.7 ys | 56,7 |
| 2012 | Morina, N | Health care utilization, somatic and mental health distress, and well-being among widowed and non-widowed female survivors of war | Netherlands | Yes, some displaced, some refugees | Kosovo | 135 | f: 100 only women | f: 48,2 |
| 2012 | Vromans, L | The Multidimensional Loss Scale Validating a Cross-Cultural Instrument for Measuring Loss | Australia | Yes | Burma | 70 | m:30 f:40 | f: 34.13 (range 18–80) |
| 2011 | Morina, N. | War and Bereavement: Consequences for Mental and Physical Distress | Netherlands | Yes, some displaced | Kosovo | 179 | m:75,  f: 104 | bereaved: 20.3 |
| 2011 | Nickerson, A | The Familial Influence of Loss and Trauma on Refugee Mental Health: A Multilevel Path Analysis | Australia | Resettled Mandean Iraqi refugees | Iraq | n=247 bereaved, n=143 (42) traumatic loss | m: 50 f: 50 | 37.7 |
| 2010 | Schaal, J | Rates and risks for prolonged grief disorder in a sample of orphaned and widowed genocide survivors | Germany | No, but conflict zone. Rwandan adult orphans/widows | Rwanda | 400 (194 widows/204 orphans) | f: 87.7 m:12.3 | 37.1 |
| 2010 | Morina, N | Prolonged Grief Disorder, Deoression, and osttraumatic Stress Disorder among bereaved Kosovar civilian war survivors: A preliminary investigation | Germany | No, but living in a post-conflict zone | Kosovo | 60 | m: 66,6 (40) f:33,3 (20) | 40.6 |
| 2010 | Silove, D | Adult Separation Anxiety Disorder Among War-Affected Bosnian Refugees: Comorbidity with PTSD and Associations with Dimensions of Trauma | Australia | Bosnian refugees | Bosnia | 126 | m: 39 f: 61 | 47 |
| 2010 | Powell, S | Missing or Killed The Differential Effect on Mental Health in Women in Bosnia and Herzegovina of the Confirmed or Unconfirmed Loss of their Husbands | Bosnia | No, but living in a post-conflict zone | Bosnia | 112 | f:100 only widows/wives whose husbands are missing | 38,2 |
| 2008 | Craig, C | Complicated Grief and Its Relationship to Mental Health and Well-Being Among Bosnian Refugees After Resettlement in the United States: Implications for Practice, Policy, and Research | USA | Refugees | Bosnia | 126 | F:56, M: 44 | 42 |
| 2004 | Momartin, S | Complicated grief in Bosnian refugees: Associations with posttraumatic stress disorder and depression | Australia | Yes | Bosnia | 126 | f:77 (61) m: 49 (39) | 47 |
| 2002 | Prigerson, H | Rates and risks of complicated grief among psychiatric patients in Karachi, Pakistan | USA | No, but in post-conflict zone | Pakistan | 151 | f: 53 m: 47 | 34 .9 |
