# Supplementary material for: The Assessment of Grief in Refugees and Post-conflict Survivors: A Narrative Review of Etic and Emic Research
Source: Front Psychol. 2018 Oct 22;9:1957. doi: 10.3389/fpsyg.2018.01957 (PMC6204364; doi:10.3389/fpsyg.2018.01957)
Supplement: Supplementary file 2 [file Table_2.docx]

| **Date** | **First author** | **Time since loss at time of measure-ment (e.g.>6m)** | **Type of bereavment** | **Sample Size** | **Grief measure (incl.cultural adaptation)** | **Prevalence of grief (incl. symptom endorsement)** | **Cultural specific adaptations (e.g. different meanings or phrases used)** | **Psychometric properties (e.g. construct validity and Crohnbach-Alpha/reliability)** |
| --- | --- | --- | --- | --- | --- | --- | --- | --- |
| 2017 | Kokou-Kpolou, K | > 3 years 51.4%, <3 years 48.6% | unexpected 66.2%, expected 33.8% | 74 | Inventory of complicated grief 17 items, with two additional questions about guilt, Idiosyncratic death and ritual information | Mean ICG-17 score migrants: 31.33, refugees 40.20, guilt feelings mean: 2,15 migrants, 2.85 refugees | Two items added: prescemce of guilt feelings related to death, and open question reasons for guilt | ANOVA comparing CG reactions between migrants and refugees |
| 2017 | Tay, A. K. | before 1975:13.8%, 1975-1999: 26.9. around 2002: 14%, 5 years ago : 26.4%, less than 6 months 8.4% | 99% traumatic loss, war related, deprivation of medical care, violence during conflict | 2964 | Idiosyncratic 4 question scale with 5 points, based on ICD-11 Maercker 2013, | including persistent yearnings or longing for  the deceased (n = 2178, 73.5%), feelings of bitterness  about the death (n = 1293, 43.6%), and feelings of emptiness  (n = 1152, 38.9%). In addition, a third (n = 957,  32.3%) reported functional impairment associated  with grief symptoms. | piloting with local people revealed 3 common items of traumatic loss, yearning, bitterness, emptiness, functional impairement was added | The items exhibited  a high level of internal reliability (α = 0.87) in the  present sample. |
| 2017 | Heeke, C | 12.4 ys | deceased n (%) 231 (75.0) disappeared n (%) 77 (25.0) | 308 | PG-13 translated into Spanish | A predominately PGD class (25.3%) | Spanish translation | For prolonged grief, the high distress class showed the highest mean (M = 45.41), Multinomial logistic regression |
| 2017 | Silove, DM | loss either 6m/5ys/post-conflict/prior 1975 | unknown,but possibly related to conflict | 1354 (677dyads) | Item pool derived from common descriptions of grief: diagnosis & classification of disorders associated with stress: proposals for ICD-11 (Maercker et al. 2013). | Total grief prevalence not available. Persistent yearnings/longings m69.3 f78.6 Bitterness about the death m35.3 f53.8 Feelings of emptiness m35.5 f45.8 Functional impairment m25.6 f40.2 | All measures translated in Tetum;field testing items of common symptoms in community | Internal reliability:0,87(satisfactory) |
| 2016 | Tay, AK | > 12 m | unclear, but a lot of traumatic losses | 230 | DSM-5 PCBD checklist and ICD-11 proposed symptoms | Total prevalence not available, intense yearnings/longings for the deceased (38.7); feeling very sorrow and pain concerning the death (40.9 %); ruminations about the deceased (33.9 %) and preoccupations with the possible causes surrounding the death (33.5 %); and difficulties reminiscing about the deceased (24.8 %). | "Duka Cita": construct of loss similar to PGD. Feelings of confusion, diminished sense of identity, difficult planning for future. | Pool of items: high level of internal reliability: KR20 of 0.94. Confirmatory factor analysis |
| 2015 | Heeke, C | unclear,but loss during conflict | death or disappearance of at least one loved one | total: 295 (73 with disappeared loved one vs. 222 bereaved) | PG-13 (Prigerson et al., 2009). PGD was translated into Spanish | Total PGD: 29.5% (n=17 23% for disappeared vs. N=70 31.5% for bereaved). PG-13 Items | PG-13 was translated into Spanish | The internal consistency of the PG-13 in this study was Alpha = 0.89. |
| 2014 | Higson-Smith, C | 5 ys or prior and ongoing | violence, sickness, accidents | 85 | None as such, but CG symptoms emerged from (semi-structured) interviews and case reviews | Confusion about role in life or diminished sense of self; difficulty accepting the loss; avoidance of reminders of the reality of the loss; inability to trust others since the loss; bitterness or anger relating to the loss; difficulty moving on with life; absence of emotion since the loss; feeling that life is empty or meaningless since the loss; and feeling stunned or shocked by the loss, functional impairment. | Interviews in French/English (selected only participants with the necessary language skills) |  |
| 2014 | Hall, BJ | unknown | unknown, but all subjects were exposed to torture | 96 | ITG (Prigerson et al., 1995) or ITG revisited (Prigerson et al. 1999) suggesting overlap Kurdish & Western conceptualizations of traumatic grief. | TG: 8,89% Sample items are “Feeling drawn to places and things associated with people who have died” and “Feeling that you have lost your sense of control.” associated with people who have died” and “Feeling that you have lost your sense of control | One symptom was specific to the Kurdish sample: “Imitating some of the same behaviors or characteristics of people who have died.” | Cronbach’s α was 0.81 |
| 2014 | Nickerson, A | unknown | unclear, but a lot of traumatic losses | 248 | Inventory of Complicated Grief (Prigerson 95) | PGD total: 17%. Longing or yearning 56.85%  Difficulty accepting death 37.10%  Difficulty trusting others 17.34%  Bitterness 45.97%  Difficulty moving on 16.13%  Numbing 11.69%  Emptiness 20.16%  No meaning 12.50% Jumpy 28.63% | All measures were translated into Arabic | Latent class analysis: PTSD/PGD class (16%), predominantly PTSD class (25%), predominantly PGD class (16%), resilient class (43%). |
| 2013b | Hinton, Nickerson | 28,9ys | unknown,but 69% of the index losses occurred during the Pol Pot period | 100 | PG-13 plus add.item:reincarnation concern.   Culturally Sensitive Measure of Grief-related distress (CSM-G):one item about somatic symptoms | PGD total: 8% (m: 25%f:75%). Functional impairment:59%  Severity score of 4 or 5 on this item (whether the person has missed  the deceased in the last month to the point of feeling poorly in the mind or  body) is considered sufficient for caseness, then 31% of the sample met criteria for complicated bereavement. Rebirth concerns/Avoidance of reminders | All measures translated in Khmer. Emic: nuk sreunoh, meaning “to recall with nostalgic longing" PG-13 plus Add. item: reincarnation concern.  Culturally Sensitive Measure of Grief-related distress (CSM-G):one item about somatic symptoms | Multivariate regression analyses with PG and CSM-G ->see table 3/4 |
| 2013a | Hinton, Field | 28,9 ys | loss in PP period | 100 | PG-13 and Dream frequency scales. Translated into Khmer | PG-13 items and Dreams: 52% percent of participants had dreamed of a deceased relative in the last month, 28% had at least two such dreams in the last month (Table 1), and 75% of participants had dreamed of a deceased relative in the last 12 months | Dreaming of the deceased scale included | Good internal consistency (a¼.94), test–retest reliability (r¼.80), and construct validity |
| 2013c | Hinton, Peou | participants were at least 6ys old at the start of PP period | loss due to genocide | 100 | (DSM-5; Shear et al. 2011), including physical symptoms and dreams. Semi-structured interview | 76% pained recall of the deceased (73% were crying when having it, 70% had a khya'l atttack) | Cambodians believe somatic symptoms are caused by a disturbance of the flow of khyaˆl, a wind-like substance thought to flow alongside blood |  |
| 2013 | Stammel, N | 30ys | unclear,but bereavment during KR regime | 775 | ICG-R (Prigerson et al., 2008) not validated for use in Cambodia, but translated into Khmer | PGD total: 111 (14,3%). Symptoms: Longing and yearning, trouble accepting death, inability trusting people, bitterness or anger related to death, uneasy about moving on, numbness/detachment, feeling empty or meaningless, bleak future, felt on edge/jumpy/easily startled | ICG-R translated into Khmer | Multiple regression analysis: Only gender significant on PGD |
| 2012 | Morina, N | A decade post war | war related loss | Bereaved n= 135 total: 206. Widowed mothers(100),non-bereaved married mothers (71),bereaved married mothers (35) | PGD-I (Interview by Prigerson 95) (based on the ICG) Albanian version | PGD 69%. ICG Items | PGD-I translated into Albanian | PGD-I:Internal consistency: Alpha=0,76. x² tests, t-tests, ANCOVA were used |
| 2012 | Vromans, L | unknown | unclear, but probably conflict-related | 70 | Multidimensional Loss Scale (MLS) (Vromans 2012). Grief questionnaire by Steel (personal communication, 2007) | Loss distress: 39% (family member) 26% (friend). E.g. (1) Do memories of the person(s) often come into your mind? (4) Do reminders (such as photos, conversations or music) often bring memories into your mind? (5) Since the loss(es) or separations(s), have you felt particularly lonely, empty inside, or socially withdrawn? | All participants required interpreters | Cronbach alphas: satisfactory internal consistency for Experience of Loss Events (0.85) and Loss Distress (0.92), reflecting a unitary construct of multidimensional loss |
| 2011 | Morina, N. | A decade post war | war related loss | bereaved: 179  total: 354 | PG-13 (Prigerson 2009) | PG-13 Items | Unmentioned, but probably PG-13 translated into Albanian | x2 tests, t-tests and multivariate logistic regression analyses |
| 2011 | Nickerson, A | unknown | 42% of all bereaved exprecienced violent losses | 247 | The Inventory of Complicated Grief is a 12-item measure assessing symptoms (Prigerson et al., 1995) | ICG Grief M=2.40 SD=1.07. Complicated grief, including longing and yearning for the deceased, difficulty moving on, difficulty accepting the death, a sense of emptiness, feeling as though the future has no meaning or purpose, and related impairment | ICG translated into Arabic | Good internal consistency (α=.94), test-retest reliability (r =.80), and construct validity with measures of depression, grief & quality of life |
| 2010 | Schaal, J | 12ys | 70% violent losses | 400 | PG -13 (Prigerson et al., 2009) | PGD 8%. PG-13 Items (percents available) | PG-13 translated into Kinyarwanda | Multiple Regression analysis, PGD as dependant variable |
| 2010 | Morina, N | 7ys | people who lost 1st-degree relatives in war | 60 | ICG-r; Prigerson & Jacobs, 2009) | PGD total: 38,3% | ICR-r translated into Albanian | ICG-r: internal consistency α = .80. Logistic regression analyses: only gender significant to PGD |
| 2010 | Silove, D | unknown | unknown | 126 | The Core Bereavement Items (Burnett, Middleton, Raphael, & Martinek, 1997), 17 items | Complicated grief: 30%. images and thoughts (of the lost person), acute separation (yearning and searching for the person in familiar places), and grief (feelings of sadness, loneliness, and longing). | Interpreter was present | Logistic regression analysis |
| 2010 | Powell, S | 6-9 years before the date of the interview (March 2001) | husbands either missing or was killed during war | 112 | UCLA/BYU Expanded Grief Inventory (Layne et al 2001) | No prevalence found. loading strongest on normal grief factor: ‘‘I miss my husband’’ and ‘‘I feel sad about his death/going missing.’’ Strongest loading on existential grief factor: ‘‘I feel more lonely since he died/went missing’’ and ‘‘I feel that my life is empty without him.’’ Traumatic grief factor best described by ‘‘I don’t do nice things that I want or need to do because they remind me of him’’ and ‘‘Life for me doesn’t have much purpose since he died/went missing.’’ | UCLA already existed in Bosnian language only for adolescents, but the simple language suited the participants often weak literacy level | ANOVAs with conﬁrmed versus unconﬁrmed loss as factor, and stressors and symptom scores as dependent variable |
| 2008 | Craig, C | NA | 80 unexpected death of loved one | 126 | ICG translated into Bosnian 19 item | 54% Prolonged Grief Disorder | back translation and consultation with cultural expert |  |
| 2004 | Momartin, S | 5ys (2-7ys) | unclear,but a lot of traumatic losses,all during war | 126 | The CBI (=Core Bereavment Items, Burnett et al, 1997) CG symptoms: 17 items, subscales: (1) images and thoughts (2) acute separation (3) grief | 39% images and thoughts about deceased, 18% acute separation anxiety 26% specific symptoms of grief. First subscale: intrusive images, unwanted thoughts, and the distress associated with reminders of the lost person. Second subscale: five items dealing with separation phenomena e.g. yearning, pining, and searching for the person in familiar places. Third subscale: five items including feelings of sadness, loneliness, and longing | CBI translated into Bosnian | Alpha coefficient of CBI was .91, signifying a high level of internal consistency.41 |
| 2002 | Prigerson, H | 5.3 months | various (48% violent losses) | 151 | Early version of ICG 7R, ITG Prigerson et al., 2001) | PGD 34%. (see symptom list Prigerson 02) | ITG translated into Urdu | ANOVA models: CG symptom score means by mode of death and relationship to the deceased, t tests significant |
